# CircAge: A Comprehensive Resource for Aging-associated Circular RNAs Across Species and Tissues

**DOI:** 10.1093/gpbjnl/qzaf044

**Published:** 2025-05-12

**Authors:** Xin Dong, Zhen Zhou, Yanan Wang, Ayesha Nisar, Shaoyan Pu, Longbao Lv, Yijiang Li, Xuemei Lu, Yonghan He

**Affiliations:** State Key Laboratory of Genetic Evolution & Animal Models, Key Laboratory of Healthy Aging Research of Yunnan Province, Kunming Institute of Zoology, Chinese Academy of Sciences, Kunming 650201, China; State Key Laboratory of Genetic Evolution & Animal Models, Key Laboratory of Healthy Aging Research of Yunnan Province, Kunming Institute of Zoology, Chinese Academy of Sciences, Kunming 650201, China; University of Chinese Academy of Sciences, Beijing 100049, China; State Key Laboratory of Genetic Evolution & Animal Models, Key Laboratory of Healthy Aging Research of Yunnan Province, Kunming Institute of Zoology, Chinese Academy of Sciences, Kunming 650201, China; University of Chinese Academy of Sciences, Beijing 100049, China; Biodiversity Data Center of Kunming Institute of Zoology, Chinese Academy of Sciences, Kunming 650201, China; Yunnan Key Laboratory of Biodiversity Information, Kunming Institute of Zoology, Chinese Academy of Sciences, Kunming 650201, China; State Key Laboratory of Genetic Evolution & Animal Models, Key Laboratory of Healthy Aging Research of Yunnan Province, Kunming Institute of Zoology, Chinese Academy of Sciences, Kunming 650201, China; University of Chinese Academy of Sciences, Beijing 100049, China; State Key Laboratory of Genetic Evolution & Animal Models, Key Laboratory of Healthy Aging Research of Yunnan Province, Kunming Institute of Zoology, Chinese Academy of Sciences, Kunming 650201, China; Biodiversity Data Center of Kunming Institute of Zoology, Chinese Academy of Sciences, Kunming 650201, China; Yunnan Key Laboratory of Biodiversity Information, Kunming Institute of Zoology, Chinese Academy of Sciences, Kunming 650201, China; Laboratory Animal Center, Kunming Institute of Zoology, Chinese Academy of Sciences, Kunming 650201, China; National Resource Center for Non-Human Primates, Kunming Institute of Zoology, Chinese Academy of Sciences, Kunming 650201, China; Laboratory Animal Center, Kunming Institute of Zoology, Chinese Academy of Sciences, Kunming 650201, China; National Resource Center for Non-Human Primates, Kunming Institute of Zoology, Chinese Academy of Sciences, Kunming 650201, China; State Key Laboratory of Genetic Evolution & Animal Models, Key Laboratory of Healthy Aging Research of Yunnan Province, Kunming Institute of Zoology, Chinese Academy of Sciences, Kunming 650201, China; University of Chinese Academy of Sciences, Beijing 100049, China; Biodiversity Data Center of Kunming Institute of Zoology, Chinese Academy of Sciences, Kunming 650201, China; Yunnan Key Laboratory of Biodiversity Information, Kunming Institute of Zoology, Chinese Academy of Sciences, Kunming 650201, China; State Key Laboratory of Genetic Evolution & Animal Models, Key Laboratory of Healthy Aging Research of Yunnan Province, Kunming Institute of Zoology, Chinese Academy of Sciences, Kunming 650201, China; University of Chinese Academy of Sciences, Beijing 100049, China

**Keywords:** Aging, circRNA, Database, miRNA, RNA-binding protein

## Abstract

Circular RNAs (circRNAs) represent a novel class of RNA molecules characterized by a circular structure and enhanced stability. Emerging evidence indicates that circRNAs play pivotal regulatory roles in the aging process. However, a systematic resource that integrates aging-associated circRNA data remains lacking. Therefore, we developed a comprehensive database, CircAge, which encompasses 756 aging-related samples from 7 species and 24 tissue types. Through high-throughput sequencing, we also generated 47 new tissue samples from mice and rhesus monkeys. By integrating predictions from multiple bioinformatics tools, we identified over 529,856 unique circRNAs. Our data analysis revealed a general increase in circRNA expression levels with age, with approximately 23% of circRNAs demonstrating sequence conservation across species. The CircAge database systematically predicts potential interactions between circRNAs, microRNAs (miRNAs), and RNA-binding proteins (RBPs), and assesses the coding potential of circRNAs. This resource lays a foundation for elucidating the regulatory mechanisms of circRNAs in aging. As a comprehensive repository of aging-associated circRNAs, CircAge will significantly accelerate research in this field, facilitating the discovery of novel biomarkers and therapeutic targets for aging biology and supporting the development of diagnostic and therapeutic strategies for aging and age-related diseases. CircAge is publicly available at https://circage.kiz.ac.cn.

## Graphical abstract

**Figure:**
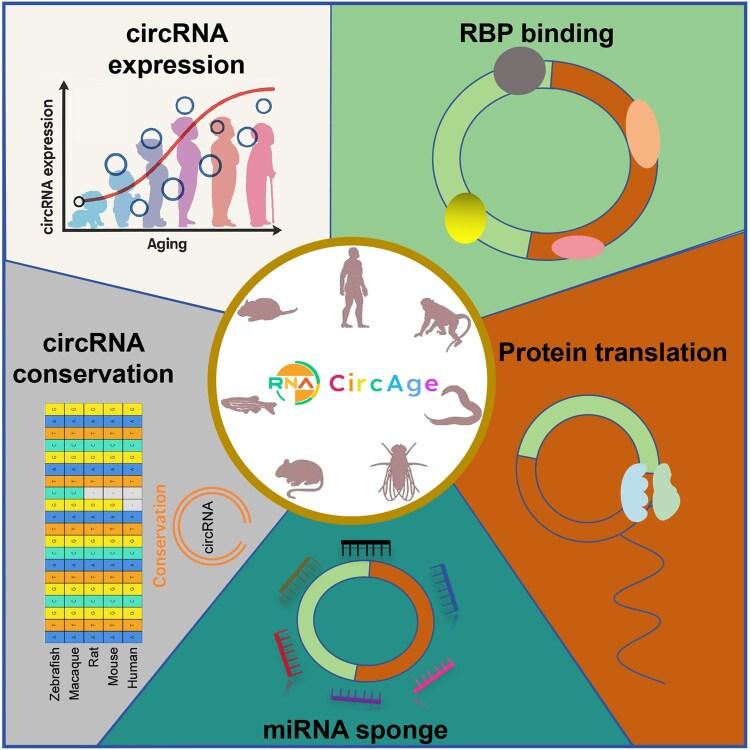

## Introduction

Circular RNAs (circRNAs) are a novel class of RNA molecules characterized by a circular structure, exhibiting higher stability and cell-type-specific expression compared to linear RNAs [1,2]. Recent studies have demonstrated that circRNAs not only participate in gene expression regulation but also play pivotal roles in crucial biological processes such as cell differentiation, proliferation, and apoptosis [3–6]. With advancements in aging research, accumulating evidence suggests that circRNAs exert critical regulatory functions during the aging process. Research has revealed that circRNAs progressively accumulate in aging tissues, including the heart and brain [7,8]. circRNAs can modulate aging and age-related diseases through diverse mechanisms. For instance, circHIPK3 acts as a scaffold for HuR and E3 ubiquitin ligase, promoting HuR degradation and consequently influencing cardiac aging [9]. circ-Foxo3 can bind to aging-associated proteins such as ID-1, E2F1, and FAK, thereby promoting cellular senescence [10]. However, only a limited number of studies have elucidated the mechanisms by which circRNAs contribute to aging. The specific roles and modes of action of most circRNAs in aging and age-related diseases remain largely unexplored. Therefore, there is an urgent need to establish a comprehensive database of aging-associated circRNAs to facilitate research in this field.

Currently, several public circRNA databases have been established, such as circBase [11], which have cataloged circRNA sequences and annotations from numerous species. Additionally, databases like cancer-specific circRNA database 2.0 (CSCD2) [12], Tissue-Specific CircRNA Database (TSCD) [13], and MiOncoCirc [14] have collected circRNA expression and function information from normal and cancer samples, providing valuable resources for investigating the roles of circRNAs in tumorigenesis and other diseases. However, no database has systematically collected aging-associated samples and identified aging-associated circRNAs, presenting an inconvenience for research in this field.

Therefore, the establishment of a systematic database for aging-associated circRNAs, which integrates expression profiles across diverse tissues and age groups while predicting potential regulatory mechanisms, will provide an invaluable resource for comprehensively understanding the roles of circRNAs in aging. This database will not only aid in the discovery of novel aging biomarkers and therapeutic targets but also foster interdisciplinary collaborative research. By offering new perspectives for the diagnosis and treatment of aging and age-related diseases, it will significantly advance both basic and clinical translational research on circRNAs within the field of aging biology.

## Data collection and processing

### Data sources

We comprehensively collected aging-related tissue and cell line sequencing data from the Gene Expression Omnibus (GEO) database and Genome Sequence Archive (GSA) database [15]. Specifically, we searched the two databases with keywords like “circRNA AND aging”, “RNA-seq AND aging”, or “RNA-seq AND senescence”. Datasets with both aging and young samples were included in the analysis. Further, we screened for samples that were prepared using total RNA with ribosomal RNA (rRNA) depletion, making them suitable for identifying aging-associated circRNAs. We obtained samples from 24 tissue types across 7 species: *Homo sapiens*, *Macaca mulatta*, *Mus musculus*, *Rattus norvegicus*, *Drosophila melanogaster*, *Caenorhabditis elegans*, and *Danio rerio*, totaling 756 samples.

**Figure 1:**
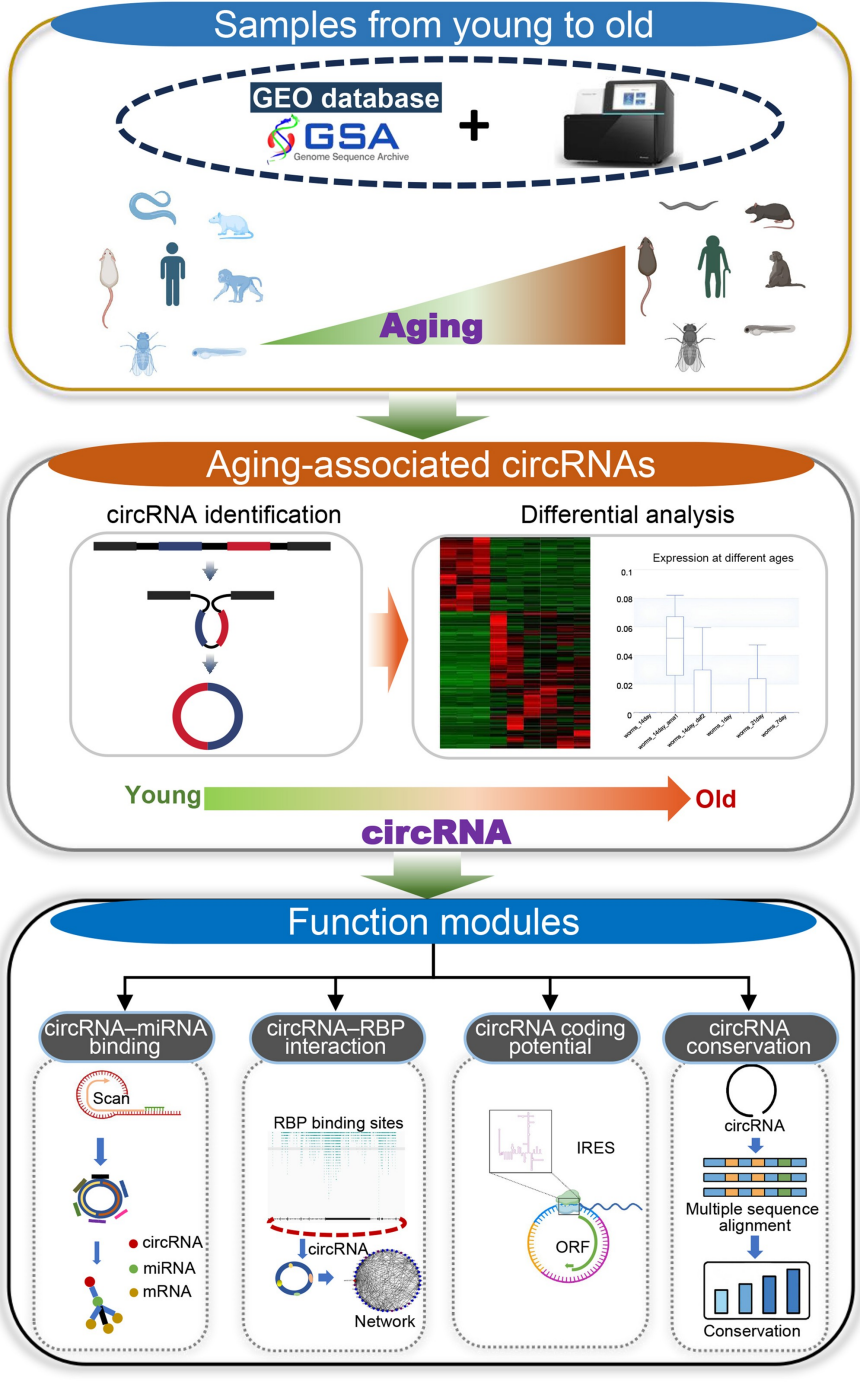
Schematic of the data processing pipeline in the CircAge database The CircAge database collects multi-species data from the GEO and GSA databases and large-scale sequencing in lab. The CircAge combines two classic identification tools to detect circRNAs in each sample, and performs differential analysis combined with age information to screen for aging-associated circRNAs. The CircAge performs annotation and sequence extraction for all circRNAs by combining gene annotations and conducts systematic functional predictions such as circRNA–miRNA binding, circRNA–RBP interaction, circRNA coding potential, and circRNA conservation. circRNA, circular RNA; miRNA, microRNA; RBP, RNA-binding protein; GEO, Gene Expression Omnibus; GSA, Genome Sequence Archive; mRNA, messenger RNA; IRES, internal ribosome entry site; ORF, open reading frame.

To further expand our database with young and aging tissue samples from mice and rhesus monkeys, we conducted high-throughput sequencing. We collected age-gradient (young, middle-aged, and old) samples of fat, heart, kidney, lung, and spleen tissues from mice, as well as liver and spleen tissues from rhesus monkeys, totaling 47 samples. The rRNA depletion RNA sequencing (RNA-seq) libraries were prepared for sequencing using the Illumina HiSeq 6000 platform by BioLinker Technology (Kunming, China). By accumulating aging data across multiple species and systemically predicting the expression and functions of circRNAs, we aim to comprehensively elucidate the molecular dynamics underlying the aging process (**Figure 1**).

### Tissue sequencing

Total RNA was extracted from mouse and rhesus monkey tissues, followed by rRNA depletion. The remaining RNA was then reverse-transcribed into complementary DNA (cDNA) and constructed into strand-specific libraries. High-throughput sequencing was performed on these libraries using the Illumina NovaSeq 6000 system.

### circRNA identification

To remove sequencing adapters and low-quality bases, quality control of the raw reads was performed using Trim Galore (https://github.com/FelixKrueger/TrimGalore). The clean reads were mapped to the reference genome using the spliced transcripts alignment to a reference (STAR) software [16]. CIRCexplorer2 [17] and circRNA_finder [18] were employed for circRNA identification and quantification. We integrated the predictions from both algorithms and normalized the circRNA back-spliced junction (BSJ) counts using the counts per million (CPM) method, calculated as the number of circRNA BSJs × 10^6^ divided by the number of mapped reads. Additionally, we annotated the circRNAs with their host genes using in-house scripts and genomic annotation files. Based on the genomic locations, circRNAs can be classified as exonic circRNAs, intronic circRNAs, or intergenic circRNAs. Furthermore, circRNAs were categorized as originating from messenger RNAs (mRNAs) or long noncoding RNAs (lncRNAs), depending on whether their parental genes were protein-coding or not.

### Identification of aging-associated circRNAs

For each tissue, we combined the normalized circRNA expression levels into a matrix. Utilizing the grouping information, we performed differential expression analysis with Student’s *t*-test. circRNAs exhibiting significant differential expression (*P* ≤ 0.05) between age groups were classified as aging-associated circRNAs.

### circRNA conservation analysis

The CircAge database encompasses circRNA data from multiple species. To assess the conservation of these circRNAs, we first reconstructed their full-length sequences using previously established methods [11]. Then, we extracted a 100-bp window (± 50 bp) surrounding the back-splice site of each circRNA, and performed basic local alignment search tool (BLAST) searches to compare the junction sequences across tissues and species [19]. Subsequently, we filtered the alignment results, retaining those with an E-value ≤ 1 × 10^−5^, identity > 80%, and an alignment length > 80 bp. circRNAs with matches in multiple species were considered conserved.

### circRNA–microRNA binding prediction

To provide potential microRNA (miRNA) binding sites within the circRNA sequences, we scanned the full-length circRNA sequences using the miRanda and TargetScan algorithms to obtain all potential miRNA binding locations [20,21]. Additionally, to facilitate downstream target prediction for users, we integrated validated miRNA–target gene data from the TarBase database [22], allowing users to conveniently query circRNA–miRNA–target gene interaction networks. Furthermore, to enable users to explore the downstream functions of miRNAs, we performed Gene Ontology (GO) enrichment analysis on all target genes with the clusterProfiler package [23].

### circRNA–RNA-binding protein binding prediction

We obtained crosslinking-immunoprecipitation and high-throughput sequencing (CLIP-seq) data for 4 species (*H*. *sapiens*, *M*. *mulatta*, *D*. *melanogaster*, and *C*. *elegans*) from the POSTAR3 database [24]. Using these data, we predicted potential RNA-binding proteins (RBPs) that may interact with circRNAs based on previous methods [25], retaining RBP binding sites within the circRNA sequences or 1 kb upstream or downstream regions.

### circRNA coding potential prediction

circRNAs have been shown to encode functional peptides. In CircAge, we comprehensively predicted the coding potential of all circRNAs. We employed the coding potential assessment tool (CPAT) algorithm to assess the coding potential of circRNA sequences and further used IRESfinder to detect the presence of internal ribosome entry sites (IRESs) within the circRNA sequences [26–28].

### Database construction

The CircAge database was deployed in a virtual machine running Windows Server 2016 using Internet Information Services (IIS) for the web service. The backend of the CircAge database was developed using C# and the Active Server Page.NET Model-View-Controller (MVC) framework, meanwhile the web frontend was organized using HyperText Markup Language (HTML), Cascading Style Sheets (CSS), JavaScript, and Layui framework. All those data were stored in the Microsoft Structured Query Language (SQL) Server. Given the massive data volume of over 200 million entries, Apache Solr was implemented to enable blazing-fast searches. MinIO, an S3-compatible object storage system, provided the associated files for downloading. The interactive visualization of the analysis results was implemented using jQuery, Apache ECharts (https://echarts.apache.org/), and D3.js (https://d3js.org/).

## Database content and usage

### Overview of CircAge database

**Figure 2:**
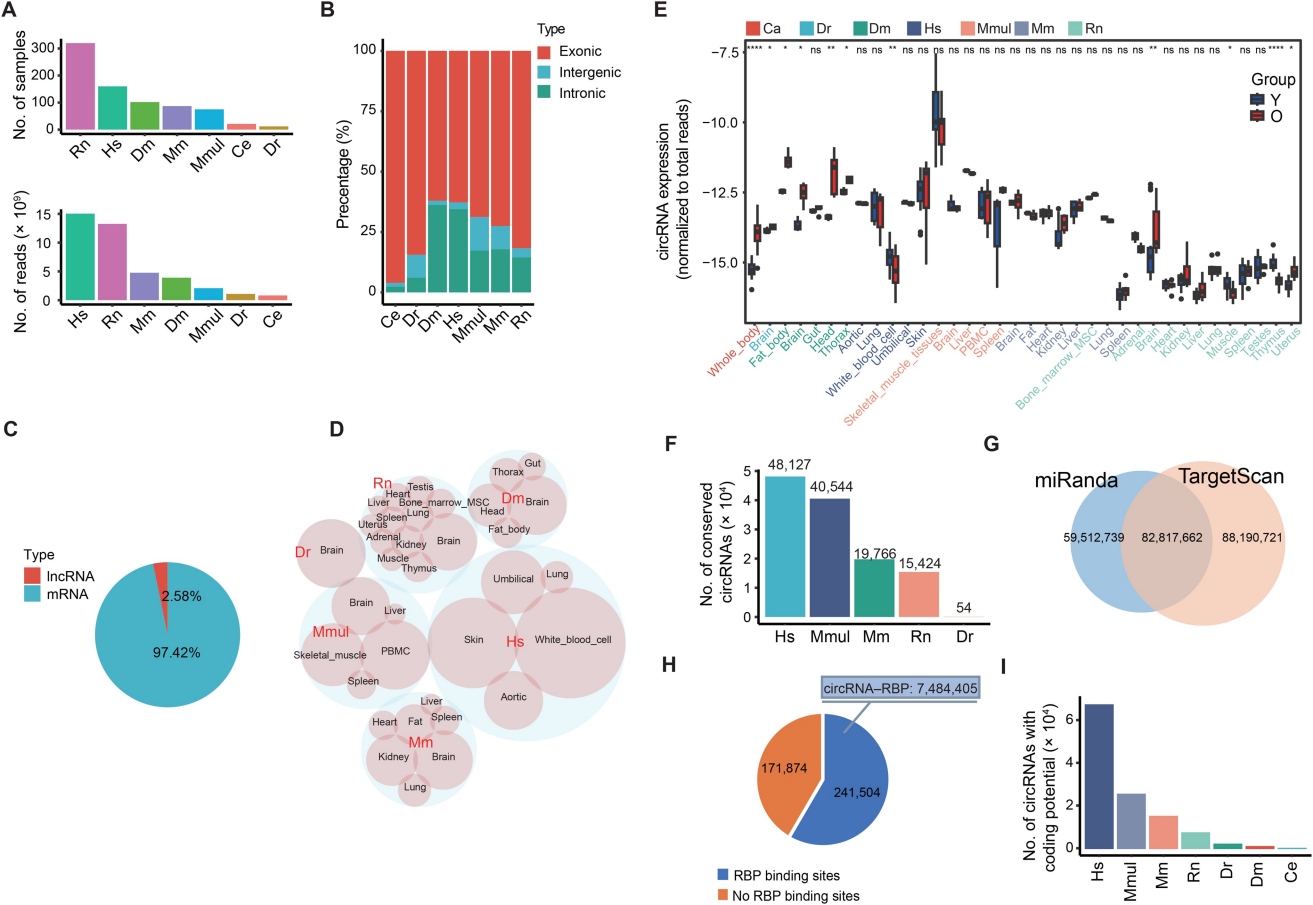
Data statistics and summary of the CircAge database **A**. Number of collected samples and sequencing reads in each species. **B**. Proportion of circRNAs in different genomic locations. **C**. Proportion of circRNAs in different host gene types. **D**. Number of circRNAs detected in various species and tissues, with larger bubbles representing higher abundance. **E**. Overall circRNA expression (normalized to total reads) profiles across different species and tissues. *, *P* ≤ 0.05; **, *P* < 0.01; ***, *P* < 0.001; ****, *P* < 0.0001; ns, not significant (Student’s *t*-test). **F**. Number of conserved circRNAs in different species. **G**. Number of miRNA binding sites in circRNAs predicted by miRanda and TargetScan. **H**. Number of RBP binding sites in circRNAs, with the upper annotation indicating the total number of circRNA–RBP interactions. **I**. Number of circRNAs with coding potential in different species. Hs, *Homo sapiens*; Mm, *Mus musculus*; Mmul, *Macaca mulatta*; Rn, *Rattus norvegicus*; Dm, *Drosophila melanogaster*; Dr, *Danio rerio*; Ce, *Caenorhabditis elegans*; lncRNA, long noncoding RNA; PBMC, peripheral blood mononuclear cell; MSC, mesenchymal stem cell.

We collected a total of 756 aging-related samples from multiple GEO and GSA datasets, encompassing 7 species (*H*. *sapiens*, *M*. *mulatta*, *M*. *musculus*, *R*. *norvegicus*, *D*. *melanogaster*, *C*. *elegans*, and *D*. *rerio*) and 24 tissue types (Table S1). Additionally, we generated 47 new RNA-seq samples, including tissues (fat, heart, kidney, lung, and spleen) from young, middle-aged, and old mice, as well as rhesus monkey liver and spleen samples. After quality control, we obtained 30 billion raw reads, with an average of 37 million reads per sample (**Figure 2**A).

By integrating the circRNA predictions from CIRCexplorer2 and circRNA_finder, we identified a total of 529,856 unique circRNAs across all samples. The identified circRNAs originated from different genomic regions, with 71.44% being exonic circRNAs, 23.01% intronic circRNAs, and 5.54% intergenic circRNAs (Figure 2B). Both protein-coding and non-coding genes contributed to circRNA biogenesis, with 97.42% circRNAs derived from mRNAs and 2.58% from lncRNAs (Figure 2C).

The number of detected circRNAs varied across species and tissues (Figure 2D). Overall, human samples yielded more circRNAs compared to other species. Brain tended to exhibit higher circRNA abundances. We performed differential expression analysis for circRNAs in each tissue to identify aging-associated circRNAs. In total, 25,262 circRNAs were found to be significantly associated with aging (*P* ≤ 0.05) across the 24 different tissues. circRNA expression showed an increasing trend during the aging process, especially in the brain. There is a significant cumulative trend of circRNAs in *D*. *rerio*, *D*. *melanogaster*, and *R*. *norvegicus* (Figure 2E).

To facilitate a more comprehensive analysis of interactions, we incorporated a co-expression analysis module into the CircAge database. This feature allows users to query the co-expression patterns between circRNAs and RBPs or lncRNAs, providing insights into their potential functional relevance in aging.

### circRNA conservation and functional annotation

We performed sequence alignments to identify conserved circRNAs across different species, considering circRNAs with similar sequences as conserved. The results revealed 123,915 conserved circRNAs, accounting for an average of 23% of the total circRNAs in each species (Figure 2F). This finding suggests a high degree of species-specificity for circRNAs, consistent with a previous study [25]. By incorporating phylogenetic analysis, we compared the similarity of conserved circRNAs across different species and found that species with closer evolutionary relationships, such as *H*. *sapiens* and *M*. *mulatta*, exhibit higher circRNA sequence similarity, consistent with their phylogenetic relationships (Figure S1A and B).

Furthermore, we obtained mature miRNA sequences and identity documents (IDs) from the TargetScan website and used the TargetScan and miRanda algorithms to predict miRNA binding sites within the full-length circRNA sequences. Our analysis identified a substantial number of 230,521,122 miRNA binding sites in circRNAs (Figure 2G). Additionally, we integrated the validated miRNA–target gene interactions from the TarBase database and performed GO enrichment analysis on the downstream target genes of miRNAs, facilitating users’ research. For circRNA–RBP interactions, we integrated data from the POSTAR3 database and obtained numerous RBP binding sites in circRNAs. The results indicate that most circRNAs harbor RBP binding sites (Figure 2H). Meanwhile, the CircAge database has predicted the coding potential of circRNAs, and the results show that many circRNAs have coding potential (Figure 2I).

### Web interface and usage

**Figure 3:**
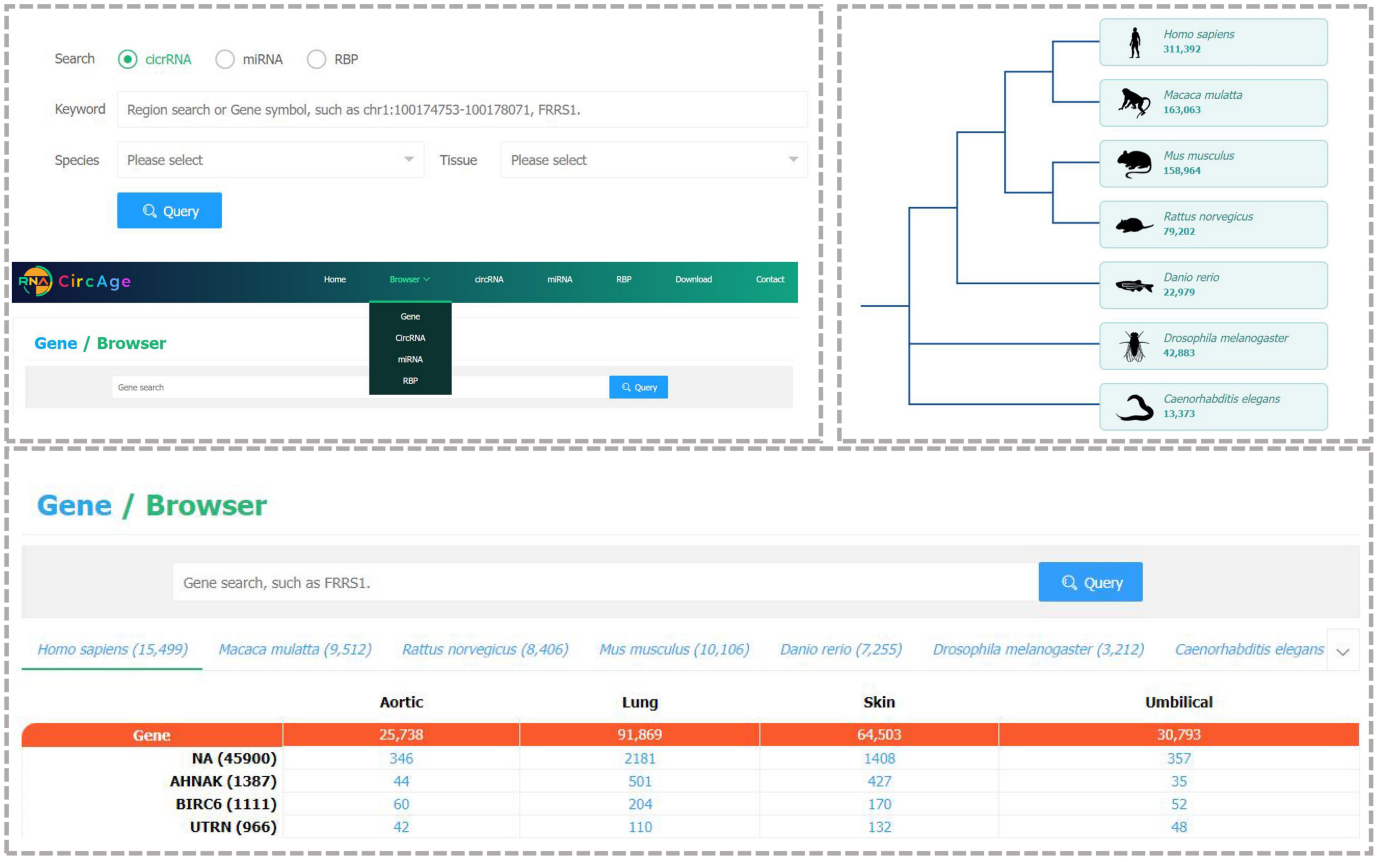
Basic functions of the CircAge database on the Home and Browser pages

CircAge provides a user-friendly web interface. The Home page mainly shows the introduction to the CircAge database, basic statistics, and data summary. Clicking on a specific species will redirect the user to the circRNA search interface for that species. Users can also perform quick searches for circRNAs, miRNAs, and RBPs on the Home page (**Figure 3**).

On the Browser page, users first need to select whether to browse the gene, circRNA, miRNA, or RBP module. The page will then generate a matrix by species, with rows representing genes, circRNAs, miRNAs, or RBPs, and columns representing tissues. The numbers in the matrix indicate the number of records for that gene, circRNA, miRNA, or RBP in the CircAge database. Clicking on a row name will navigate to the search results for that entry, while clicking on a number in the matrix will redirect to the search page for that gene, circRNA, miRNA, or RBP in the corresponding tissue (Figure 3).

**Figure 4:**
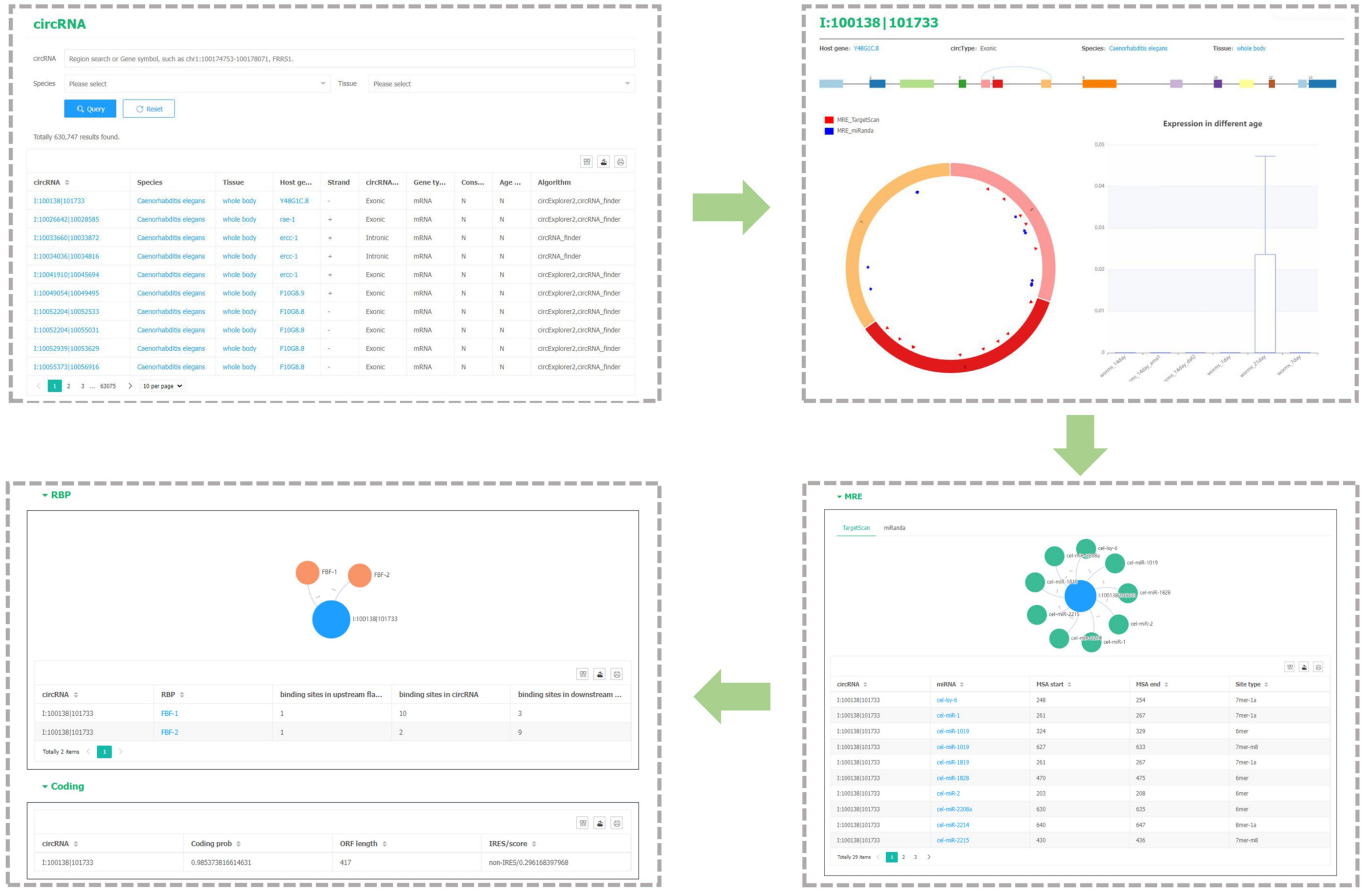
The circRNA page of the CircAge database The page displays circRNA search, circRNA annotation, circRNA structure, circRNA expression, miRNA and RBP binding sites within circRNAs, and circRNA coding potential prediction results.

The CircAge database consists of three modules: circRNA, miRNA, and RBP. On the circRNA page, users can search for circRNAs by coordinates or browse by selecting a specific species and tissue type. The table below displays detailed information about each circRNA, including its ID, species, host gene, and circRNA type. Users can click on the species, tissue, and host gene columns to perform further searches for the corresponding terms. Additionally, clicking on a circRNA entry will redirect users to its detail page, which displays the circRNA’s main information. The top section shows a schematic diagram of the circRNA, with the upper panel illustrating the structure of the host gene and the circRNA generation site, and the lower left panel displaying the circRNA structure and potential miRNA binding sites. The lower right part shows the circRNA expression levels across different age groups. Below, the page presents predicted circRNA–miRNA binding results, circRNA–RBP binding predictions, and circRNA coding potential predictions (**Figure 4**).

**Figure 5:**
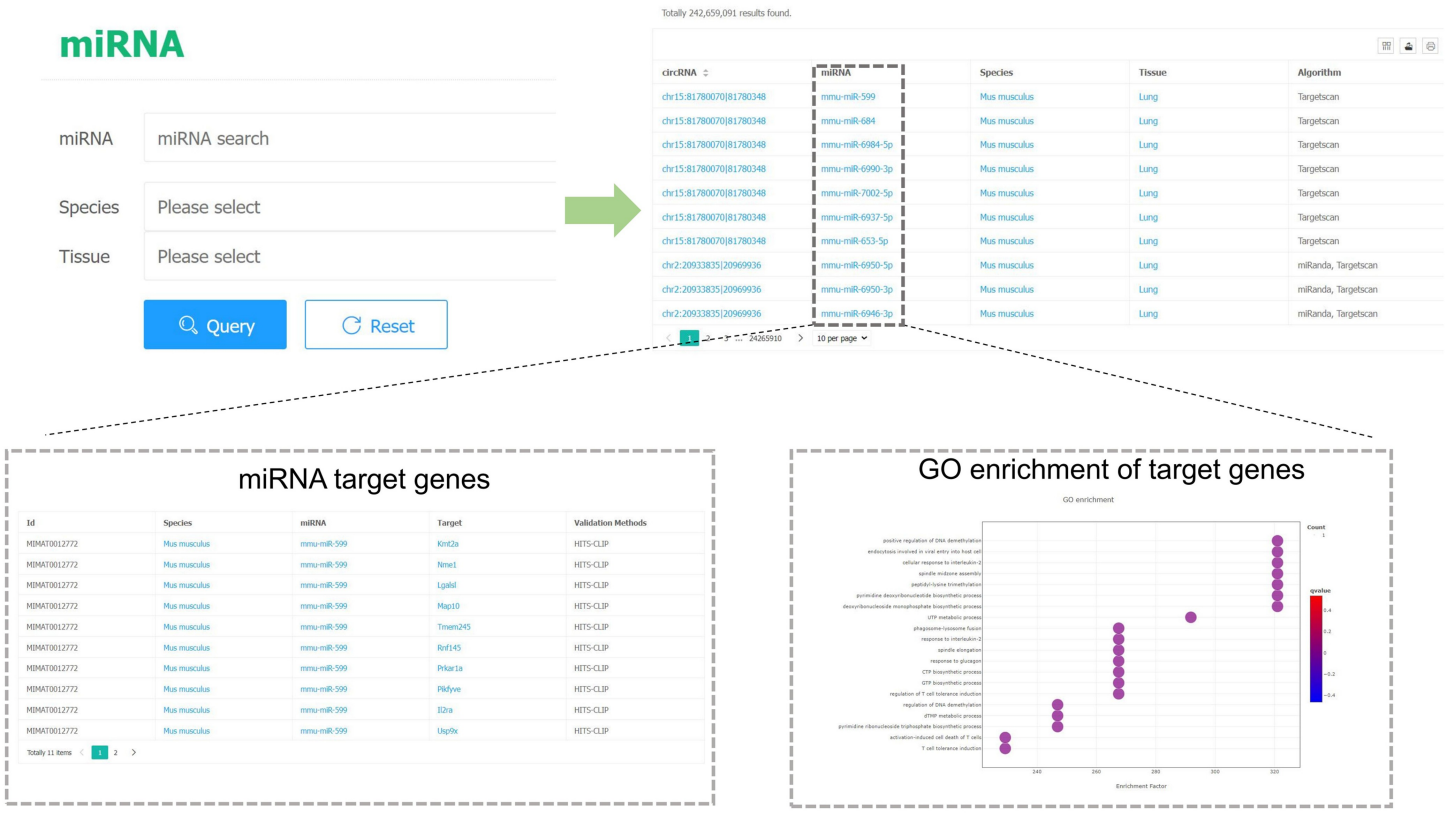
The miRNA page of the CircAge database The page provides a miRNA search function and integrates downstream target genes of miRNAs and functional analysis results of the target genes. GO, Gene Ontology.

On the miRNA page, users can search for specific miRNAs of interest. CircAge will return all circRNAs that interact with the queried miRNA. Clicking on a circRNA will display its detail page, while clicking on a species or tissue will redirect to the search interface. Clicking on a miRNA will return a list of its validated target genes and GO enrichment results, allowing users to explore the circRNA–miRNA–mRNA regulatory network and potential downstream functions (**Figure 5**).

**Figure 6:**
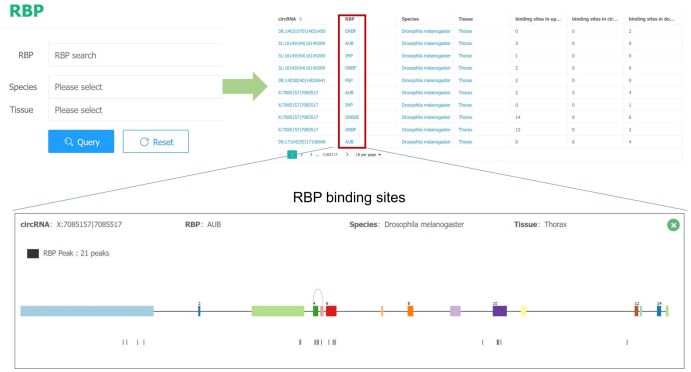
The RBP page of the CircAge database The page provides an RBP search function and allows users to view the specific binding sites of RBPs on circRNAs and genes.

On the RBP page, users can search for specific RBPs, and CircAge will return all circRNAs that bind to the queried RBP. Users can also click on the RBP column, and CircAge will display the structure of the corresponding host gene, circRNA generation site, and all binding sites for the RBP within that gene region. All circRNA information in the CircAge database is openly accessible, and users can download data through the Download page (**Figure 6**).

## Database application

To highlight the utility of the CircAge database, we searched for aging-associated circRNAs across different species. Using expression trend analyses, we identified significant aging-associated circRNAs, including circCCNB1 and circHIPK3 in *H*. *sapiens* and circGRIA1 in *M*. *mulatta*. The CircAge database offers functional predictions, such as miRNA and RBP binding sites, which enable the construction of circRNA regulatory networks.

**Figure 7:**
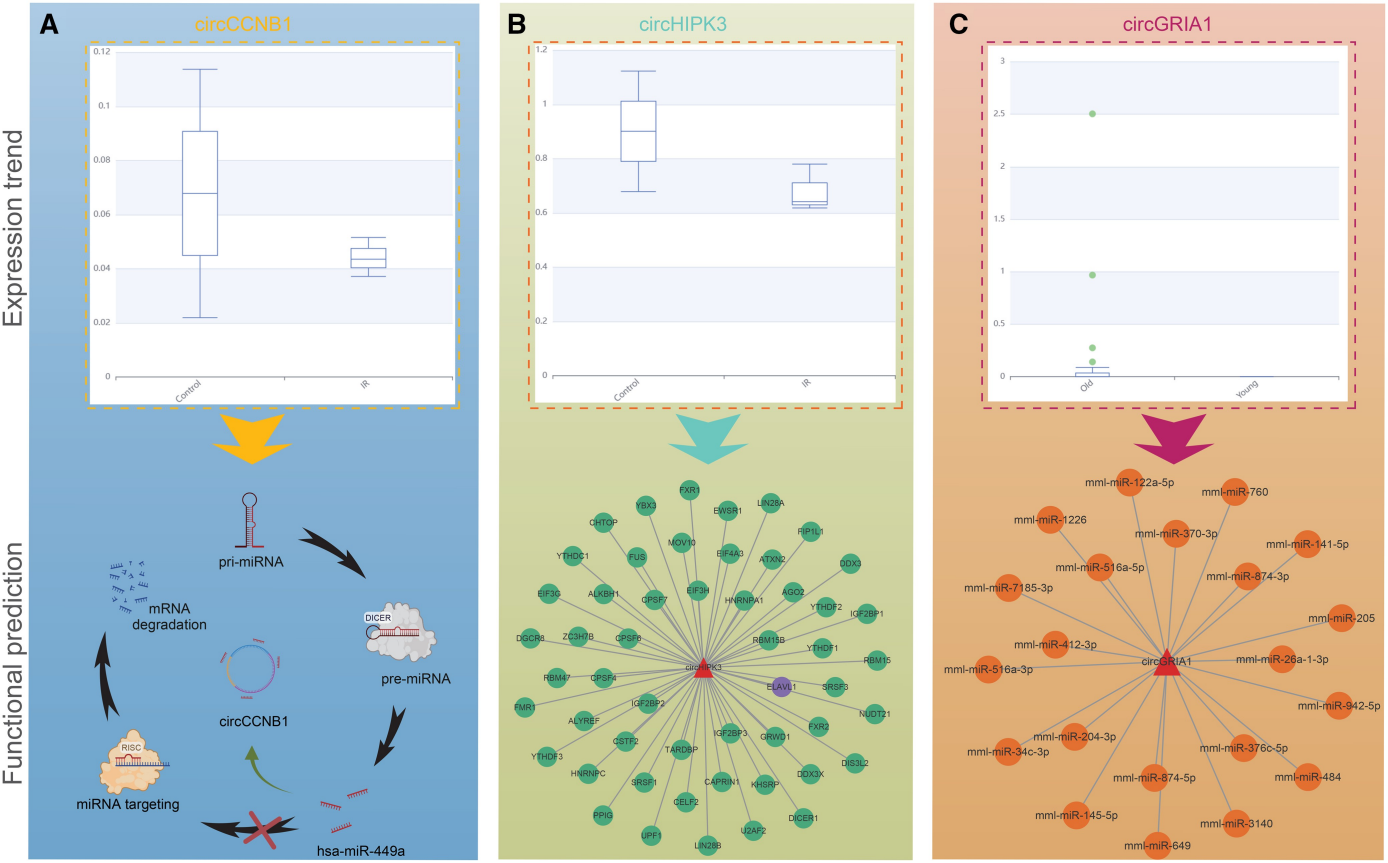
CircAge usage example **A**. The circCCNB1 expression trend in the CircAge database and the circCCNB1–hsa-miR-449a–*CCNE2* axis predicted by CircAge has been validated to play a role in the regulation of aging [29]. **B**. The circHIPK3 expression trend in the CircAge database and its RBP binding sites. **C**. The circGRIA1 expression trend in the CircAge database and its miRNA binding sites.

For instance, detailed analysis revealed that circCCNB1 originates from exons 6 and 7 of the *CCNB1* gene and contains multiple miRNA binding sites. By integrating circRNA–miRNA and miRNA–target gene interaction data from CircAge, we constructed a comprehensive regulatory network for circCCNB1. A literature review confirmed that the anti-aging function of circCCNB1 has been experimentally validated (**Figure 7**A). It functions as a molecular sponge for hsa-miR-449a, thereby alleviating this miRNA’s repressive effect on its target *CCNE2* and consequently inhibiting cellular senescence [29].

Furthermore, circHIPK3 exhibits significant downregulation during aging, and CircAge predicts its potential interactions with multiple RBPs. Literature evidence confirmed an experimentally validated interaction between circHIPK3 and HuR, wherein circHIPK3 functions as a protein scaffold that mediates HuR ubiquitination and degradation, thereby modulating cardiac aging processes (Figure 7B) [9].

Finally, recent studies have demonstrated that circGRIA1 is upregulated in the aging macaque brain, a finding consistent with expression trends documented in the CircAge database [30]. circGRIA1 modulates synaptic plasticity and synaptogenesis in the macaque brain and displays a negative correlation with its host gene expression. While the precise regulatory mechanism remains to be elucidated, the competing endogenous RNA pathway represents a classical mechanism through which circRNAs exert their functions. The CircAge database enables the prediction of circRNA–miRNA interactions, providing valuable insights into circGRIA1’s miRNA sponging potential and facilitating future mechanistic investigations (Figure 7C).

## Discussion and conclusion

In this study, we established a comprehensive database of aging-associated circRNAs, providing a critical resource for investigating the regulatory roles of circRNAs during the aging process. The CircAge database encompasses data from multiple species and tissue types, and systematically integrates circRNA expression profiles, conservation information, potential interactions with miRNAs and RBPs, and coding potential, laying the foundation for elucidating the functions of circRNAs in aging biology. Within the database, circRNA expression levels exhibit an overall upward trend with increasing age, suggesting that circRNAs may be broadly involved in regulating the aging process. Approximately 23% of circRNAs exhibit sequence conservation across different species, and these conserved circRNAs warrant focused attention and in-depth investigation, aiding in the elucidation of conserved regulatory mechanisms underlying the aging process.

The CircAge database continuously integrates the latest research developments, creating an extensive circRNA knowledge base that facilitates the elucidation of the complex relationship between circRNAs and aging. Compared to existing circRNA databases, CircAge is the only database for aging research with a large amount of data and systematic prediction of circRNA function (Table S2). The inception of CircAge holds substantial significance, expediting research into the regulatory mechanisms of circRNAs within aging biology and serving as a reference for investigating circRNA functions in other biological processes. In the future, with further experimental validation and complementary studies, the pivotal roles of circRNAs in aging and associated diseases will be further elucidated. This will establish a foundation for the development of advanced diagnostic and therapeutic strategies.

## Ethical statement

This study was approved by the Institutional Animal Care and Use Committee of the Kunming Institute of Zoology, Chinese Academy of Sciences (Approval No. IACUC-PA-2023-08-031).

## Supplementary Material

qzaf044_Supplementary_Data

## Data Availability

CircAge is freely available at https://circage.kiz.ac.cn/. All RNA-seq raw data have been deposited in the Genome Sequence Archive [15] at the National Genomics Data Center (NGDC), China National Center for Bioinformation (CNCB) (GSA: CRA016736), and are publicly accessible at https://ngdc.cncb.ac.cn/gsa. CircAge has been submitted to Database Commons [31] at the NGDC, CNCB, which is publicly accessible at https://ngdc.cncb.ac.cn/databasecommons/database/id/9701.

## CRediT author statement

**Xin Dong:** Methodology, Validation, Formal analysis, Data curation, Writing – original draft, Funding acquisition. **Zhen Zhou:** Methodology, Validation, Investigation, Writing – review & editing. **Yanan Wang:** Software, Formal analysis, Data curation, Writing – review & editing. **Ayesha Nisar:** Writing – review & editing. **Shaoyan Pu:** Software, Data curation. **Longbao Lv:** Supervision, Resources. **Yijiang Li:** Resources. **Xuemei Lu:** Software, Resources, Visualization, Project administration. **Yonghan He:** Conceptualization, Supervision, Resources, Project administration, Funding acquisition. All authors have read and approved the final manuscript.
