# Supplementary figures and images for: CircAge: A Comprehensive Resource for Aging-associated Circular RNAs Across Species and Tissues
Source: Genomics Proteomics Bioinformatics. 2025 May 12;23(3):qzaf044. doi: 10.1093/gpbjnl/qzaf044 (PMC12448220; doi:10.1093/gpbjnl/qzaf044)

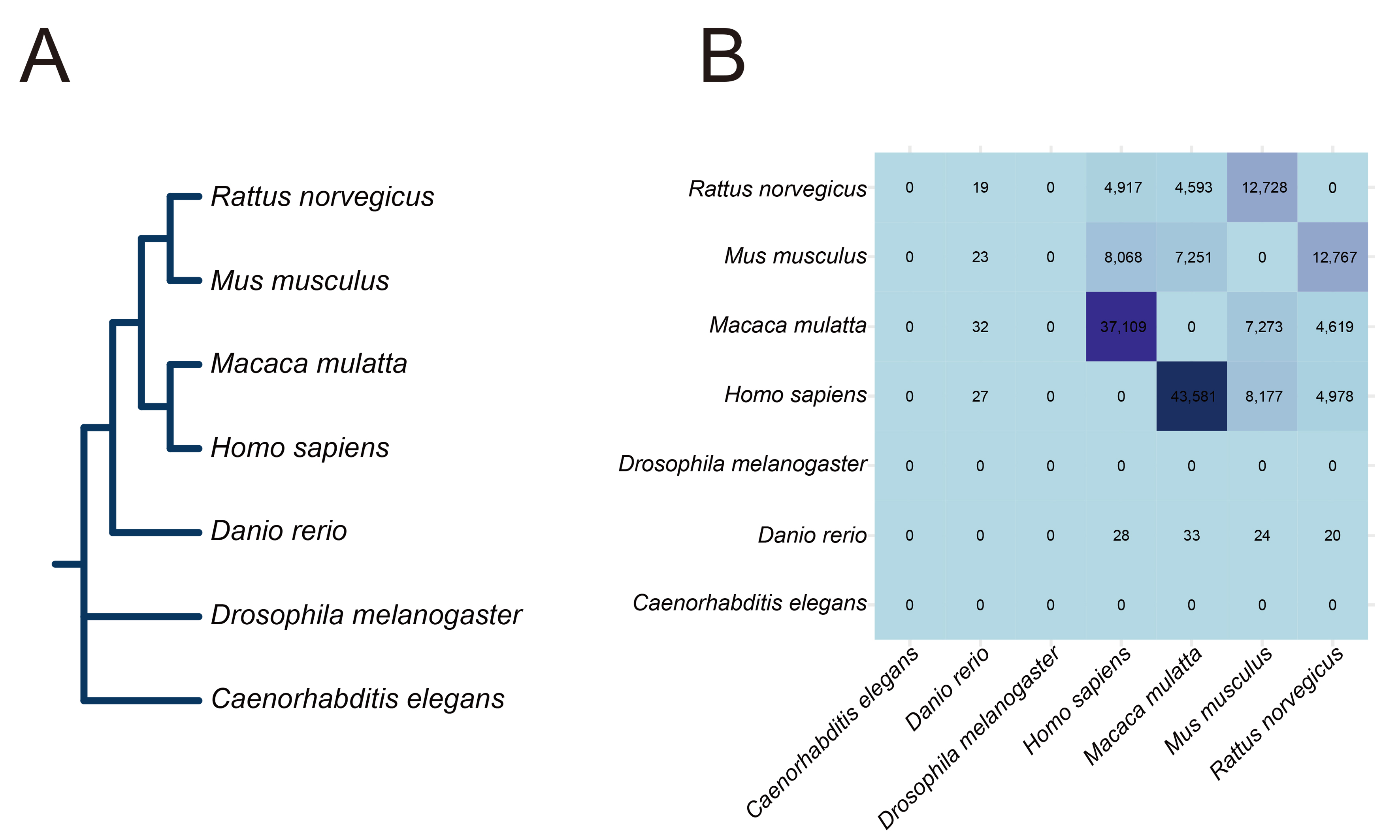

Supplement: qzaf044_Supplementary_Data [file qzaf044_supplementary_data.zip › Figure S1.jpg]
