# Supplementary material for: CircAge: A Comprehensive Resource for Aging-associated Circular RNAs Across Species and Tissues
Source: Genomics Proteomics Bioinformatics. 2025 May 12;23(3):qzaf044. doi: 10.1093/gpbjnl/qzaf044 (PMC12448220; doi:10.1093/gpbjnl/qzaf044)
Supplement: qzaf044_Supplementary_Data [file qzaf044_supplementary_data.zip › Supplementary_Data.zip!/Supplementary material captions.docx]

**Supplementary materials**

**Figure S1 Phylogenetic analysis of species and circRNA conservation analysis**

A. Phylogenetic tree of species in the CircAge database. **B**. Heatmap displaying the number of circRNAs with high sequence similarity across species in the CircAge database.

**Table S1 Sample informations and accessions of CircAge database**

**Table S2 Comparison of CircAge with circAtlas 2.0, circBase, CircRic, CSCD, and CSCD2**
